# Bioorthogonal Chemical Ligation Creates Synthetic Antibodies with Improved Therapeutic Potency

**DOI:** 10.1021/acscentsci.3c00240

**Published:** 2023-03-13

**Authors:** Ruixiang Wang, Peng Zou

**Affiliations:** † Academy for Advanced Interdisciplinary Studies, PKU-Tsinghua Center for Life Science, Peking University, Beijing 100871, China; ‡ College of Chemistry and Molecular Engineering, Academy for Advanced Interdisciplinary Studies, PKU-Tsinghua Center for Life Science, Synthetic and Functional Biomolecules Center, Beijing National Laboratory for Molecular Sciences, Key Laboratory of Bioorganic Chemistry and Molecular Engineering of Ministry of Education, PKU-IDG/McGovern Institute for Brain Research, Peking University, Beijing 100871, China, Chinese Institute for Brain Research (CIBR), Beijing, 102206, China; § Chinese Institute for Brain Research (CIBR), Beijing 102206, China

Antibodies, also known as immunoglobulins, are proteins secreted by immune cells to specifically bind foreign antigens, often from invading species. Immunoglobulin G (IgG), one of the major classes of antibodies, is composed of two heavy chains and two light chains that are interlinked via disulfide bonds, resulting in two identical fragment antigen-binding (Fab) domains for recognizing biological targets, and one fragment crystallizable (Fc) domain that mediates immune reaction and improves stability. In recent years, chemically producing and/or modifying antibodies for improved therapeutic functions have attracted attention across the fields of chemistry and medicine. In this issue of *ACS Central Science*, Thoreau et al. report a bioorthogonal chemical ligation technique to construct synthetic antibodies, dubbed SynAbs.^1^ The overall structure of SynAbs mimics the naturally occurring IgG, but they differ in one key aspect: unlike IgGs that specifically recognize only one epitope per antibody, SynAbs can be made to contain two distinct antigen-binding sites, enabling them to bind two biological targets at a time. In other words, they are bispecific.

Bispecific antibodies (bsAbs) are attractive as they are capable of recruiting immune cells to attack tumors or increasing binding specificity. Owing to recent advances in antibody conjugation methodologies, more than 200 types of bsAbs have been made for clinical and preclinical trials.^2^ However, many of these synthetic strategies are incapable of incorporating the Fc domain that could confer bsAbs with higher stability, better solubility, longer half-life,^3^ and enhancement of tumor killing capacity due to Fc-mediated antibody-dependent cell-mediated cytotoxicity (ADCC) and complement dependent cytotoxicity (CDC) effects.^4^ Existing methods for producing Fc-containing IgG-like bsAbs are almost exclusively protein bioengineering, including quadroma cell line technology,^5^ “knobs-into-holes”,^6^ CrossMab,^7^ etc. Despite great progress made by these strategies, bioengineering methods are often hampered by low yield, complicated purification processes, and low modularity.

**Figure 1:**
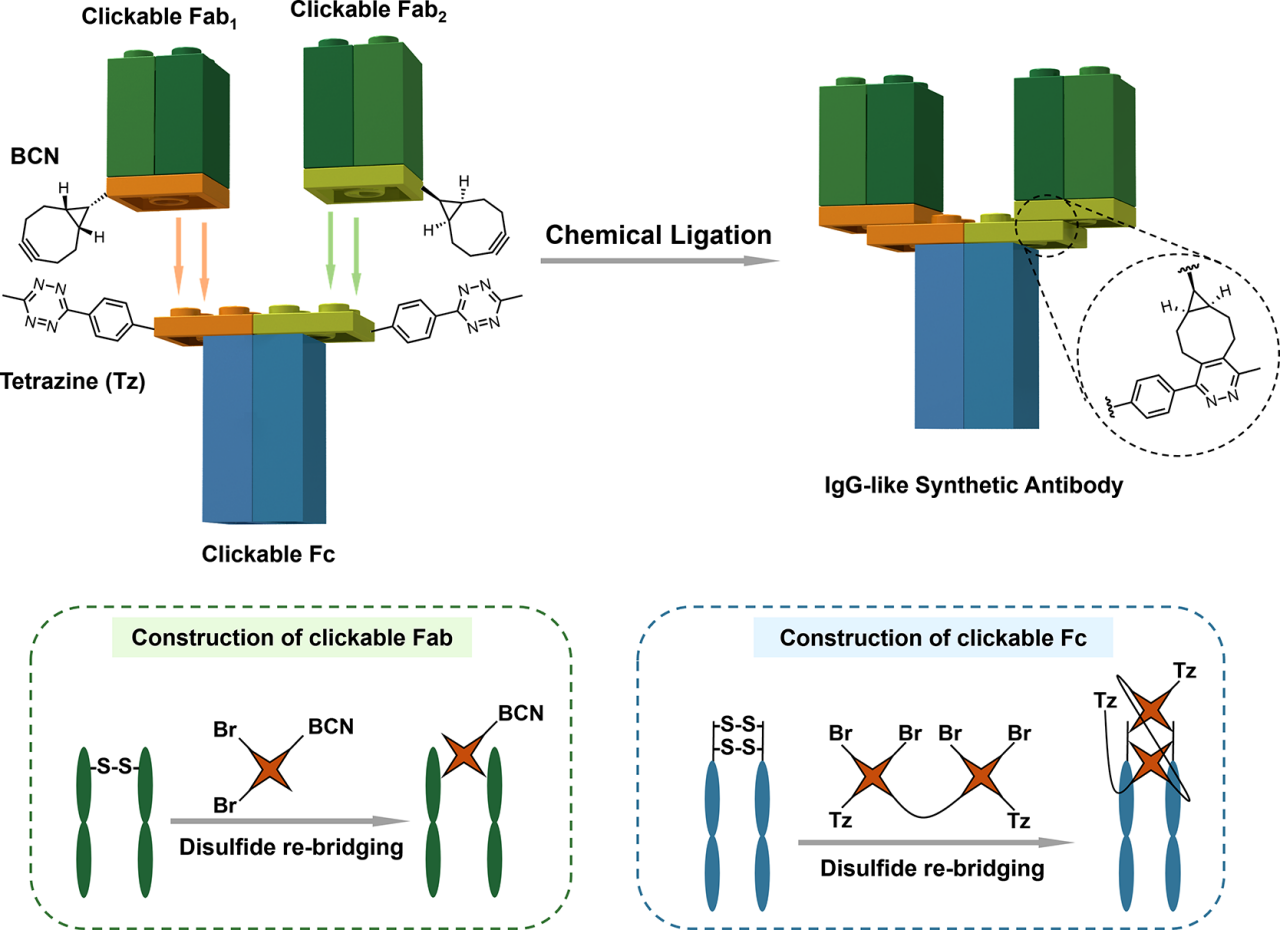
Modular chemical ligation of IgG-like bivalent SynAbs. Constructing IgG-like SynAbs requires clickable-Fab and clickable-Fc as the building blocks. With the help of site-selective disulfide rebridging reactions, a bicyclononyne (BCN) group was incorporated to Fab, while two equivalents of tetrazine (Tz) groups were incorporated to Fc, and then the Fc-(Fab_1_)-Fab_2_ format SynAbs could be assembled through BCN-Tz click reactions.

In this study, the authors developed a pure chemical ligation method to generate Fc-containing antibodies based on disulfide rebridging and click chemistry. Previous studies reported a subset of cysteine reactive disulfide rebridging reagents, which could reduce disulfide bridges and then covalently rebridge the cysteines via small molecules. By adding click handles to the reagents, protein–protein conjugates could be easily generated through click chemistry.^8,9^ To construct IgG-like bsAbs, the authors started by producing HER2/HER2 bivalent monospecific antibodies. After the site-selective disulfide rebridging reaction of CD20 Fc and HER2 Fab fragments, the dually clickable-Fc and mono clickable-Fab could be generated to assemble Fc_CD20_-(Fab_HER2_)_2_ antibodies through a tetrazine-bicyclononyne (BCN) click reaction. The strategies described by the authors achieved first-in-class purely chemical construction of full IgG-like bivalent antibodies (Figure 1), which are very modular and efficient.

As a proof-of-concept demonstration, the HER2/CD3 bispecific SynAb was used as a bispecific T cell engager (BiTE). The Fc domain of CD20 was functionalized using disulfide rebridging reagents with two equivalent tetrazine handles, so the Fab_HER2_ and Fab_CD3_ could be introduced sequentially to generate a Fc_CD20_-(Fab_HER2_)-Fab_CD3_ construct. Incubating the resulting bispecific SynAb with T cells and epithelial carcinoma HCC1954 cells led to T cell activation and HCC1954 cell death, which demonstrates its capacity to recruit T cells to attack the target cells.

The reported strategy is highly modular and easy to industrialize: both Fc and Fab fragments were obtained from commercial mAbs, and the production of one SynAb was accomplished within 5 days with high yield. It is anticipated that this novel approach could be further applied to generate an array of IgG-like antibodies for biomedical and clinical investigations. Future improvement of the method could benefit from a greater choice of bioorthogonal chemical ligations. For example, if the disulfide rebridging reagents contained two distinct click handles, antibody assembly processes could be well controlled in a more orthogonal manner. Additionally, the Fc domains could be carefully chosen and further modified to fully unlock the potential of SynAbs, for example: (1) SynAbs containing an Fc domain are expected to have a longer half-life, so introducing a suitable Fc domain to currently developed non-IgG bsAbs promises to increase their serum half-life. (2) Tumor killing capacity could be enhanced through Fc-mediated ADCC and CDC effects; this property could be utilized to improve therapeutic potency. (3) The Fc domain could be further modified to generate antibody-drug conjugates for cancer treatment. (4) The click handle bearing the Fc domain could be used as a platform to attach multiple molecules, such as cytokines or metabolites, for extended functionality. We expect the functions of Fc-containing SynAbs will be fully exploited, and bioorthogonal chemical conjugation methods will drive technological progress in immune therapy.
